# Effects of sodium houttuyfonate on transcriptome of *Pseudomonas aeruginosa*

**DOI:** 10.1186/s13104-019-4721-2

**Published:** 2019-10-22

**Authors:** Yeye Zhao, Yuanqing Si, Longfei Mei, Jiadi Wu, Jing Shao, Changzhong Wang, Daqiang Wu

**Affiliations:** 1 Department of Pathogenic Biology and Immunology, College of Integrated Chinese and Western Medicine, Anhui University of Chinese Medicine, Hefei, 230012 China; 2 Key Laboratory of Xin’An Medicine, Ministry of Education, Anhui University of Chinese Medicine, Hefei, 230038 China

**Keywords:** RNA-seq, *Pseudomonas aeruginosa*, Sodium houttuyfonate, Transcriptome

## Abstract

**Objectives:**

The purpose of this experiment is to analyze the changes of transcriptome in *Pseudomonas aeruginosa* under the action of sodium houttuyfonate (SH) to reveal the possible mechanism of SH inhibiting *P. aeruginosa*. We analyzed these data in order to compare the transcriptomic differences of *P. aeruginosa* in SH treatment and blank control groups.

**Data description:**

In this project, RNA-seq of BGISEQ-500 platform was used to sequence the transcriptome of *P. aeruginosa*, and sequencing data of 8 samples of *P. aeruginosa* are generated as follows: SH treatment (SH1, SH2, SH3, SH4), negative control (Control 1, Control 2, Control 3, Control 4). Quality control is carried out on raw reads to determine whether the sequencing data is suitable for subsequent analysis. Totally 170.53 MB of transcriptome sequencing data is obtained. Then the filtered clean reads are aligned and compared to the reference genome to proceed second quality control. After completion, 5938 genes are assembled from sequencing data. Further quantitative analysis of genes and screening of differentially expressed genes based on gene expression level reveals that there are 2047 significantly differentially expressed genes under SH treatment, including 368 up-regulated genes and 1679 down-regulated genes.

## Objective

*Pseudomonas aeruginosa* is a gram-negative bacterium, which can produce endotoxin, exotoxin, proteolytic enzyme and other substances and infect human and other organisms [1, 2]. At present, macrolide and aminoglycoside antibiotics are commonly used for curing the clinical infection of *P. aeruginosa*. However, with the emergence of drug resistance, *P. aeruginosa* are difficult to treat by common antibiotics. Thus, we are seeking for effective antimicrobial agents from traditional Chinese medicine to treat infection of *P. aeruginosa*. Previously, our research group have proved that sodium houttuyfonate (SH) can effectively inhibit the *P. aeruginosa* [3, 4]. Here, our aim is to investigate possible antimicrobial mechanism of SH by comparing the transcriptomic differences between SH drug and blank control groups.

The assemble transcriptome contains thousands of transcripts. Thus this study provides transcriptomic comparison between SH medication group and blank control group rather than comparisons of expression of several certain genes such as *algD*, *algR*, *lasI*, *phzM*, *lasA* and *bdlA*, in previous studies [4–6]. The difference of these transcriptome can be used as the basis for studying gene expression changes in SH treatment and control groups.

## Data description

We cultured *P. aeruginosa* under two conditions, with 4 biological replications which were cultured independently under each condition:

1. ATCC 27853 was inoculated into LB liquid medium and cultured overnight at 37 °C. The culture were centrifuged for 1 min at 12,000 r/min, and pour out the supernatant, diluted with sterile water to 0.5 Maxwell colorimetric tube, and diluted to 10^7^ times for later use. The SH was prepared according to our previous research [6]. The prepared SH samples in LB medium with 1 MIC (minimum inhibitory concentration) SH of 512 μg/ml were cultured for 24 h at 37 °C until OD_600_ was 0.6–0.8, and collected by centrifugation of 1 min at 12,000 r/min, and then rinsed with sterile water for 3 times. Then we placed the collected bacterial samples in the centrifuge tube, sealed it with sealing film, and sent the sample for RNA-seq with dry ice.
2. The samples of blank control were collected similarly as SH treatment samples except without drug treatment.

Totally 170.53 MB of transcriptome sequencing data is obtained after RNA-seq applying BGISEQ-500 platform. The original data of sequencing includes reads with low quality, linker contamination and high content of unknown base N are removed before data analysis to ensure the reliability of the results. This project used SOAPnuke [7], a filtering software independently developed by Huada Corporation, to make statistics and trimmomatic [8] to filter. Firstly, readers including connectors are removed. Then the reads with unknown base N content more than 5% are wiped off. Finally, the low-quality reads are removed (we define reads with a mass value of less than 10 and a proportion of more than 20% of the total number of bases in the reads as low-quality reads). The filtered “Clean Reads” are saved in FASTQ format. The file format corresponding to each sample is FASTQ format (Table 1).

**Table 1:** Overview of transcriptome data files

Label	Name of data file/data set	File types (file extension)	Data repository and identifier (DOI or accession number)
Control1	SRR9031329_1.fq	.fastq	https://www.ncbi.nlm.nih.gov/sra/SRX5808580[accn]
Control2	SRR9031328_1.fq	.fastq	https://www.ncbi.nlm.nih.gov/sra/SRX5808581[accn]
Control3	SRR9031319_1.fq	.fastq	https://www.ncbi.nlm.nih.gov/sra/SRX5808590[accn]
Control4	SRR9031318_1.fq	.fastq	https://www.ncbi.nlm.nih.gov/sra/SRX5808591[accn]
SH1	SRR9031323_1.fq	.fastq	https://www.ncbi.nlm.nih.gov/sra/SRX5808586[accn]
SH2	SRR9031322_1.fq	.fastq	https://www.ncbi.nlm.nih.gov/sra/SRX5808587[accn]
SH3	SRR9031321_1.fq	.fastq	https://www.ncbi.nlm.nih.gov/sra/SRX5808588[accn]
SH4	SRR9031320_1.fq	.fastq	https://www.ncbi.nlm.nih.gov/sra/SRX5808589[accn]
Gene expression data	GSE133428_All_samples.GeneExpression.FPKM	.txt.gz	https://www.ncbi.nlm.nih.gov/geo/query/acc.cgi?acc=GSE133428
Table S1	SH transcriptome-S1.xlsx	.xlsx	10.6084/m9.figshare.8241410.v1
Figure S1	Figure S1	.tif	10.6084/m9.figshare.8241410.v1

The raw RNA-Seq data (.fastq files) are available for download on the SRA [11]. The gene expression data (.txt.gz) are available on the GEO [12]. The additional files including Table S1 and Figure S1 can obtained on Figshare [13]

The original sequencing sequence data (fastq file), including reference genome information, can be obtained on NCBI. After the quality control of the original data, we used Bowtie2 [9] to compare clean reads to the reference gene sequence (Table S1), and then RSEM [10] was used to calculate the expression levels of genes and transcripts. After completion, totally 5938 genes are assembled from sequencing data.

In order to reflect the correlation of gene expression between samples, Pearson correlation coefficients of all gene expression amounts between every samples are calculated, and expression amount distribution analysis are performed. The obtained results are shown in Fig. S1A [13]. According to the gene expression level of each sample, a total of 2047 differentially expressed genes are detected by the threshold of fold changes > 2, Q value < 0.001, including 368 up-regulated genes and 1679 down-regulated genes. The results are shown in volcanic map of Fig. S1B [13].

## Limitations

The limitations of this data is that there is not a gradient comparison under multiple different concentrations of SH, and the transcriptome expression of *P. aeruginosa* under 24 h of culture is selected in this study, which may make the results inconsistent with other research results. In addition, in our previous studies [4, 5], the qRT-PCR results showed that genes of l*asA*, *algD*, *algR* are down regulated by SH treatment in *P. aeruginosa*. However, these genes are found to be below the detection threshold in this study. This may be due to the different technologies used in determination of the gene expression.

## Data Availability

The data described in this data description can be freely and publicly accessed on the sequence reading file (SRA) with the item number of http://identifiers.org/ncbi/insdc.sra:SRP197195 [11] and the gene expression omnibus (GEO) with the item number of https://www.ncbi.nlm.nih.gov/geo/query/acc.cgi?acc=GSE133428 [12]. We have used these data to conduct a large amount of additional analysis, and these results can be obtained from https://figshare.com/articles/SH_transcriptome-S1_xlsx/8241410 [13] and the corresponding authors according to reasonable requirements.

## Abbreviations

RNA-seq: ribonucleic acid sequencing
SH: sodium houttuyfonate
qRT-PCR: quantitative reverse transcription polymerase chain reaction
